# Always Online: Abschalten in einer mobilen Arbeitswelt

**DOI:** 10.1007/s11613-022-00770-7

**Published:** 2022-07-12

**Authors:** Kathrin Reinke, Birte Düvel

**Affiliations:** 1 Institut für Psychologie, Technische Universität Darmstadt, Alexanderstraße 10, 64289 Darmstadt, Deutschland; 2 Fachgebiet Wirtschaftspsychologie, Universität Kassel, Pfannkuchstraße 1, 34121 Kassel, Deutschland

**Keywords:** Mobiles Arbeiten, Entgrenzung der Arbeit, Abschalten, Mobile working, Boundary blurring, Detaching

## Abstract

In der heutigen mobilen Arbeitswelt verschwimmen Grenzen zwischen Arbeit und Privatleben immer mehr. Dies ist mit Vorteilen wie einer höheren Flexibilität verbunden. Auf der anderen Seite kann eine Entgrenzung der Arbeit langfristig zu Beeinträchtigungen der Gesundheit und der Leistungsfähigkeit führen. Grenzen zwischen Arbeits- und Privatleben aktiv zu gestalten, produktiv und gleichzeitig erholt zu bleiben, stellt somit eine große Herausforderung vieler Berufstätiger dar. Dieser Artikel fasst Ergebnisse aus der (Interventions‑)Forschung zur Grenzgestaltung und Erholung zusammen und zeigt Lösungsansätze für Beschäftigte und Organisationen auf, um dieser Herausforderung zu begegnen.

## Abstract (en)

In today’s mobile working world, boundaries between work and personal life domains are increasingly blurring. This offers opportunities such as greater flexibility of when and where to work. However, this boundary blurring can also impair both professionals’ health and performance in the long term. Actively managing work-life boundaries, remaining productive and recovered at the same time thus presents an important challenge for many professionals. This article summarizes research findings on (interventions for) boundary management and recovery and shows possible solutions on how employees and organizations can meet this challenge.

## Verschwimmende Grenzen zwischen Arbeit und Privatleben

In den letzten Jahren hat sich die Art und Weise, wie wir arbeiten und leben, stark verändert. Die Welt wird immer mobiler. Informations- und Kommunikationstechnologien (IKT) sorgen dafür, dass Menschen über zeitliche und physische Grenzen hinweg miteinander verbunden bleiben – und so auch unabhängig von Zeit und Ort arbeiten können. Der technologische Fortschritt und die Möglichkeit, ständig erreichbar und verbunden bleiben zu können, führen dazu, dass traditionelle Grenzen zwischen Privatem und der Arbeitswelt immer mehr verschwimmen (vgl. Boswell et al. 2016). Dieses Phänomen hat sich mit Beginn der COVID-19 Pandemie und dem Wechsel vieler Berufstätiger ins Home-Office noch verstärkt (Rudolph et al. 2021). Mehr und mehr Beschäftigte sind 24 h, sieben Tage die Woche erreichbar, um steigenden Arbeitsanforderungen wie einer erhöhten Arbeitsintensität und -geschwindigkeit gerecht zu werden.

Man spricht daher auch von einer zunehmenden „Entgrenzung der Arbeit“ (Behrens et al. 2021). Diese erhöhten Anforderungen können mit der Zeit in einer Zunahme von arbeitsbedingtem Stress münden. Zudem können durchgängige Erreichbarkeit und verschwimmende Grenzen dazu führen, dass Erholungsphasen von arbeitsbedingtem Stress verkürzt werden oder sogar komplett ausbleiben (Barber und Jenkins 2014; Wepfer et al. 2018). Das Ausbleiben von Erholung wiederum hat langfristig einen negativen Einfluss auf die physische und psychische Gesundheit von Berufstätigen – und nicht zuletzt auch auf ihre Leistungsfähigkeit (vgl. Sonnentag et al. 2017).

Die Lösung lautet in unserem digitalen Zeitalter nun sicherlich nicht, diesen Wandel der Arbeitswelt aufzuhalten und zu traditionellen Arbeitsmodellen zurückzukehren. Der aktuelle Wandel der Arbeitswelt bringt schließlich auch Vorteile mit sich wie eine höhere Flexibilität, die viele Beschäftigte sehr schätzen. Organisationen sollten sich jedoch bewusst sein, dass ihre Mitarbeitenden vor der großen Herausforderung stehen, die Grenzen zwischen Arbeits- und Privatleben aktiv zu gestalten, dabei produktiv zu arbeiten – und gleichzeitig gesund und erholt zu bleiben. Diese Herausforderung zu meistern, darf jedoch nicht nur in der Verantwortung der einzelnen Beschäftigten liegen: Die Organisations- und Teamkultur und damit einhergehende Normen und Erwartungen haben einen großen Einfluss darauf, wie Arbeitnehmende die Grenzen zwischen Arbeits- und Privatleben gestalten und inwieweit sie sich erholen können (Belkin et al. 2020; Reinke und Gerlach 2021). Zudem sind Unternehmen auch im Rahmen des Arbeitszeitgesetzes dazu verpflichtet, dass die Erholungszeiten ihrer Beschäftigten eingehalten werden. Organisationen müssen ihre Beschäftigten daher aktiv dabei unterstützen, diesen Herausforderungen erfolgreich zu begegnen.

## Grenzen zwischen Arbeit und Privatleben gestalten: Können wir noch abschalten?

### Was bedeutet Grenzgestaltung?

Menschen unterscheiden sich darin, wie sie die Grenzen zwischen Arbeits- und Privatleben gestalten (möchten). So geht die Boundary Theory (Ashforth et al. 2000) davon aus, dass sich die Präferenzen für Grenzgestaltung auf einem Kontinuum zwischen Segmentation und Integration bewegen. Personen, die eher klare Grenzen bevorzugen, werden als Segmentierende bezeichnet und präferieren es, Arbeits- und Privatrollen strikter voneinander abzugrenzen. Diese Personen arbeiten z. B. weniger gern im Home-Office, bevorzugen es, private Themen auf der Arbeit zu vermeiden und Arbeitsthemen im Feierabend hinter sich zu lassen. Werden integrierte Grenzen bevorzugt, überschneiden sich Arbeits- und Privatrollen stärker, und Grenzen zwischen Lebensbereichen werden durchlässiger gehalten. In diesem Fall wird von Integrierenden gesprochen. Diese Personen präferieren es, häufiger zwischen verschiedenen Rollen zu wechseln, und lassen Vermischungen verschiedener Lebensbereiche eher zu. Vollständige Segmentation bzw. Integration stellen dabei die Extreme auf dem Kontinuum dar. Die meisten Menschen befinden sich irgendwo dazwischen und fühlen sich „eher“ dem segmentierenden oder dem integrierenden Typ der Grenzgestaltung zugehörig (ebd.).

Die eigenen Präferenzen für die Grenzgestaltung müssen jedoch nicht immer mit der Realität, d. h. mit der tatsächlichen Grenzgestaltung, übereinstimmen. So lässt sich in den letzten Jahren – verstärkt durch die COVID-19 Pandemie – der Trend beobachten, dass Grenzen zwischen Arbeits- und Privatleben für mehr und mehr Berufstätige durchlässiger und flexibler werden und häufiger Grenzüberschreitungen stattfinden, z. B. indem Beschäftigte spätabends noch Emails lesen und beantworten, im Urlaub Anrufe entgegennehmen oder regelmäßig Arbeit mit nach Hause nehmen (Behrens et al. 2021; Boswell et al. 2016). Selbst Arbeitnehmende in „traditionelleren“ Arbeitsmodellen mit festen Arbeitszeiten und einem festen Arbeitsort bleiben mittels IKT immer häufiger außerhalb der regulären Arbeitszeiten mit der Arbeit verbunden.

Dabei ist es wichtig festzuhalten, dass die flexible Nutzung von IKT und die damit einhergehende Aufweichung klarer Grenzen zwischen Arbeits- und Privatleben zahlreiche Vorteile für Unternehmen und Beschäftigte bietet: IKT sorgen für einen schnelleren Wissensaustausch und -transfer, und Beschäftigte haben eine größere Flexibilität und Autonomie darüber, wann, wo und wie sie arbeiten. Dies kann die Vereinbarkeit von Beruf und privaten Anforderungen sogar unterstützen und zu einer größeren Zufriedenheit mit der Arbeit führen (Behrens et al. 2021; Diaz et al. 2012). Auf der anderen Seite zeigen sich jedoch auch negative Auswirkungen, insbesondere für die Erholung von Berufstätigen.

### Die Bedeutsamkeit von Erholung

Erholung ist ein lebenswichtiger Prozess, bei dem beeinträchtigte Ressourcen anhand verschiedener Aktivitäten wieder aufgebaut werden. Erholung vom Arbeitsstress findet erst dann statt, wenn Arbeitsanforderungen aufhören, einen Einfluss auf den Arbeitnehmenden auszuüben (Meijman und Mulder 1998). Erholungsprozesse spielen folglich eine wichtige Rolle für die individuelle Gesundheit und das Wohlbefinden: Sie sorgen dafür, beeinträchtigte Stimmungen und Emotionen wiederherzustellen und auch physiologische Stressreaktionen zu reduzieren (ebd.; Sonnentag und Fritz 2007). Dementsprechend kann ein Mangel an Erholung langfristig zu schwerwiegenden Beeinträchtigungen der psychischen und physischen Gesundheit wie auch der Leistungsfähigkeit führen. So zeigten Studien beispielsweise, dass sich fehlende Erholung negativ auf die Arbeitsleistung und organisationales Engagement auswirkt (s. Sonnentag et al. 2017).

Erholungsprozesse können durch unterschiedliche Erholungserfahrungen unterstützt werden. Erholungserfahrungen sind Aktionen und Aktivitäten, die einer Person dabei helfen, sich von Beeinträchtigungen durch Stress zu erholen. Sonnentag und Fritz (2007) definierten vier zentrale Erholungserfahrungen: (1) Mentales Abschalten von der Arbeit, (2) Entspannung, (3) Herausforderungen meistern (Mastery) und (4) Kontrolle in der Freizeit. Im Kontext der Entgrenzung der Arbeit und der Grenzgestaltung scheint besonders das mentale Abschalten von hoher Bedeutung zu sein (vgl. Allen et al. 2014). Mentales Abschalten meint das psychische Loslösen von der Arbeit und den damit verbundenen Arbeitsanforderungen. Beschäftigte distanzieren sich dabei folglich nicht nur körperlich, z. B. durch einen Ortswechsel vom Büro nach Hause, sondern auch mental von der Arbeit. Durch das mentale Abschalten werden arbeitsbezogene Gedanken vermieden und so zusammenhängende affektive und regulatorische Ressourcen geschont beziehungsweise neu aufgefüllt (Sonnentag und Fritz 2007). Meta-Analysen unterstützen dies und zeigen, dass mentales Abschalten einen positiven Effekt auf die Gesundheit, Lebenszufriedenheit und Schlafqualität hat sowie Erschöpfungszustände reduzieren kann (Wendsche und Lohmann-Haislah 2017).

### Wie hängt die Entgrenzung der Arbeit mit Erholung und Gesundheit zusammen?

Studien zeigen, dass ständige Erreichbarkeit in der Freizeit und verschwimmende Grenzen dafür sorgen können, dass Erholungsaktivitäten beeinträchtigt werden (Barber und Jenkins 2014; Wepfer et al. 2018). Der ständige Informationsfluss kann zu einer Informationsüberladung und zu einem Gefühl des Kontrollverlusts führen. Durch die Möglichkeit, permanent auf arbeitsbezogene Informationen zugreifen zu können, geraten Mitarbeitende, teils unbewusst, in einen Rollenkonflikt zwischen der Arbeit und dem Privatleben. Dies birgt die Gefahr, dass Arbeitnehmende sich nicht mehr mental von der Arbeit lösen und somit Erholungsprozesse nicht einsetzen können (Derks et al. 2015; Wepfer et al. 2018).

Letztendlich können diese Faktoren zu einer Beeinträchtigung des Wohlbefindens der Beschäftigten führen, z. B. indem sich der Schlaf und die Work-Life Balance verschlechtern und Erschöpfungszustände steigen (Barber und Jenkins 2014; Reinke und Gerlach 2021). Darüber hinaus zeigt die Forschung, dass die vermehrte Nutzung von IKT für Arbeitszwecke in der Freizeit und damit einhergehendes mangelndes Abschalten wichtige organisationale Erfolgsgrößen beeinträchtigt, z. B. indem das Arbeitsengagement verringert wird (Lanaj et al. 2014). Um zu verhindern, dass die Entgrenzung der Arbeit langfristig sowohl Erholungsprozesse und die Gesundheit als auch die Leistungsfähigkeit von Beschäftigten beeinträchtigt, sollten verschiedene Lösungsansätze betrachtet werden.

## Lösungsansätze für eine gesunde Grenzgestaltung und Erholung: Eine Bestandsaufnahme

### Strategien für eine aktive Grenzgestaltung

Die Forschung legt nahe, dass Berufstätige bestimmte Strategien und Praktiken nutzen können, um die Grenzen zwischen Arbeit und Privatleben aktiv und bewusst zu gestalten. Diese Praktiken werden auch Strategien der Grenzgestaltung genannt (Allen et al. 2021). Dabei wird angenommen, dass insbesondere diejenigen Strategien förderlich für die Erholung sind, die eine gewisse Segmentierung der Lebensbereiche unterstützen, da durch eine klarere Abgrenzung der Lebensbereiche z. B. Konflikte zwischen Lebensbereichen und ständige Unterbrechungen von Erholungsprozessen vermieden werden können (Rexroth et al. 2016).

Bisherige Studien zeigen, dass wichtige Strategien zur Grenzgestaltung zeitliche, physische, behaviorale und kommunikative Strategien umfassen (Allen et al. 2021; Kreiner et al. 2009). Beschäftigte können sich z. B. bewusst einen festen Arbeitsplatz im Home-Office schaffen (physische Strategie), Absprachen mit dem Team und der Führungskraft treffen, ob, wann und wie sie in ihrer Freizeit für Arbeitsbelange erreichbar sind (kommunikative Strategie), sich bewusst Zeiten blocken, in denen sie nicht arbeiten (zeitliche Strategie) oder sogenannte Übergangsrituale schaffen (behaviorale Strategie), die den Eintritt in die Arbeit oder in die Freizeit signalisieren. Die Nutzung dieser Strategien kann dabei helfen, mehr Kontrolle über die eigene Grenzgestaltung zu erlangen, Grenzüberschreitungen und Konflikte zwischen Lebensbereichen zu minimieren und mental abzuschalten (Kossek 2016).

Dabei ist jedoch zu beachten, dass die meisten Erkenntnisse zu Strategien der Grenzgestaltung aus qualitativen Studien stammen und daher wenig Rückschlüsse auf die tatsächliche Wirksamkeit einzelner Strategien für die Erholungsförderung zulassen. Folglich gibt es einen hohen Forschungsbedarf für systematische Untersuchungen der Effektivität dieser Strategien für die Grenzgestaltung und Erholung mit quantitativen, längsschnittlichen Studien (Rudolph et al. 2021).

Darüber hinaus ist die konsequente Anwendung dieser Strategien im Alltag nicht immer einfach und möglich. Sowohl Organisationen wie auch Beschäftigte müssen zunächst die Notwendigkeit dieser Strategien und der Erholungsförderung anerkennen und lernen, mit IKT und der steigenden Flexibilität gewissenhaft umzugehen, um einer Entgrenzung der Arbeit und potenziellen negativen Konsequenzen vorzubeugen (Boswell et al. 2016). Dafür braucht es entsprechende evidenzbasierte Beratungs- und Weiterbildungsmaßnahmen, in denen Organisationen, Führungskräfte und Mitarbeitende sich mit aktiver Grenzgestaltung und Erholung beschäftigen und die Anwendung dieser Strategien erlernen. Im Folgenden werden die Ergebnisse von Studien vorgestellt, die die Effektivität solcher Interventionen zur Erholungsförderung untersuchen.

### Interventionen zur Förderung der Grenzgestaltung und Erholung

In den letzten Jahren gab es mehr und mehr Studien zu Interventionen, die auf die Förderung des Wohlbefindens und der Erholung von Berufstätigen abzielen (für einen Überblick vgl. Karabinski et al. 2021; Richardson 2017). Dabei sind primäre und sekundäre Interventionen zu unterscheiden. Primäre Interventionen sind präventiv und proaktiv und setzen eher auf der Ebene der Organisation an. Sie streben systematische Änderungen der Praktiken des Unternehmens und der Arbeitstätigkeit an, z. B. indem die Reduktion von Stressoren und Änderung von Rahmenbedingungen adressiert werden. Sekundärinterventionen zielen darauf ab, Berufstätige mit Fähigkeiten und Techniken auszustatten (z. B. Erholungstechniken, Zielsetzungsstrategien), die Risiken verringern und die Stressbewältigung und Erholung verbessern. Diese Interventionen setzen eher auf der individuellen Ebene an. Interventionen zur Verbesserung der Erholung gehören mehrheitlich zu den sekundären Interventionen. Sie zielen auf die unmittelbare Verbesserung von Erholungserfahrungen ab, z. B. indem Strategien und Techniken zur Entspannung oder zum mentalen Abschalten vermittelt werden. Dabei adressieren einige Interventionsstudien auch explizit das Thema Grenzgestaltung, indem die Teilnehmenden Strategien der Grenzgestaltung erlernten (z. B. Hahn et al. 2011; Rexroth et al. 2016; für einen Überblick vgl. Karabinski et al. 2021).

Wie effektiv sind bisherige Interventionen zur Erholungsförderung nun? Eine aktuelle Meta-Analyse von 30 Interventionsstudien mit 34 Interventionen zur Verbesserung des mentalen Abschaltens zeigt vielversprechende Ergebnisse: Die Meta-Analyse konnte einen robusten, positiven Interventionseffekt für das mentale Abschalten nachweisen, welcher bis zu sechs Monate nach der Intervention anhielt (Karabinski et al. 2021). Dabei zeigte sich außerdem, dass diejenigen Interventionen, die aktive Grenzgestaltung in einem Themenbaustein adressierten, eine höhere Effektivität aufwiesen als Interventionen, die die Thematik der aktiven Grenzgestaltung nicht behandelten. Darüber hinaus weisen die Ergebnisse der Meta-Analyse darauf hin, dass Interventionen mit einer Dauer von mehr als zwei Wochen und einer Intensität von mehr als vier Trainingsstunden noch effektiver sind als kürzer andauernde und weniger intensive Interventionen. Interessant für Organisationen ist ebenfalls, dass sich angeleitete Präsenz-Interventionen und andere Formate, z. B. webbasierte Interventionen, in ihrer Effektivität nicht unterschieden (ebd.). Auch andere Interventionsstudien zum Thema Stressbewältigung konnten zeigen, dass webbasierte Interventionen effektiv Stress- und Burnout-Symptome reduzieren können (Persson Asplund et al. 2017). Im Gegensatz zu Präsenz-Interventionen können webbasierte Interventionen von überall und zu jeder Zeit durchgeführt werden. Sie sind anonym, die Teilnehmenden können in ihrem eigenen Rhythmus und Tempo teilnehmen, und sie können Mitarbeitende teils schneller und auf einer breiteren Basis erreichen, da es keine langen Wartelisten gibt. Wichtig ist jedoch, auch in webbasierten Formaten die Integration des Gelernten in den Alltag zu adressieren, um nachhaltige Verbesserungen für Erholung und Wohlbefinden zu erzielen.

## Fazit und Empfehlungen für Forschung und Praxis

Aktuelle Ergebnisse aus der Interventionsforschung sind vielversprechend: Interventionen zur Erholungsförderung scheinen wirksam zu sein und das mentale Abschalten der Teilnehmenden nachhaltig zu steigern. Dabei sind nach den Ergebnissen der Meta-Analyse von Karabinski et al. (2021) insbesondere diejenigen Interventionen effektiv, die das Thema der aktiven Grenzgestaltung miteinschließen. Dieses Ergebnis legt nahe, dass aktive Grenzgestaltung eine wichtige Rolle für Erholungsprozesse und das mentale Abschalten von Beschäftigten spielt. Dennoch hat sich die Forschung bisher selten mit der Frage befasst, welche konkreten Strategien der Grenzgestaltung besonders wichtig und effektiv für die Erholung von Berufstätigen sind und inwieweit Grenzgestaltung gezielt durch Interventionen erlernt und verbessert werden kann (Richardson 2017). Zukünftige Studien sollten folglich die Lernbarkeit und Effektivität verschiedener Strategien der Grenzgestaltung evaluieren. Auch Rahmenbedingungen in der Organisation, die die Wirksamkeit der Strategien beeinflussen könnten, sollten zukünftig stärker adressiert und erforscht werden. Zudem könnten theoretische Mechanismen der Wirksamkeit von Interventionen beleuchtet werden, die eine Umsetzung des Erlernten in den Alltag und somit die Förderung von Erholung unterstützen.

Für Unternehmen und Berufstätige lässt sich festhalten: Die Gestaltung von Grenzen stellt eine der wichtigsten Herausforderungen in der aktuellen Arbeitswelt dar (Kossek 2016). Diese Herausforderung hat durch den rasanten Anstieg von Home-Office und mobilem Arbeiten seit der Covid-19 Pandemie sogar noch an Relevanz gewonnen. Gleichzeitig zeigen Ergebnisse aus der Forschung, dass eine Entgrenzung der Arbeit langfristig zu erheblichen Beeinträchtigungen der Gesundheit und auch der Leistungsfähigkeit von Beschäftigten führen kann (Sonnentag et al. 2017; Wepfer et al. 2018). Es ist folglich erforderlich für Unternehmen, sich über Vor- und Nachteile der IKT-bedingten Verbundenheit und Entgrenzung der Arbeit klarzuwerden und gemeinsam mit den Arbeitnehmer:innenvertretungen und Gewerkschaften ihre Mitarbeitende aktiv bei der Herausforderung, Grenzen zu gestalten, produktiv und gleichzeitig erholt zu bleiben, zu unterstützen.

Wie kann dies konkret aussehen? Erste Ergebnisse aus der Interventionsforschung zeigen, dass Beschäftigte durch Interventionen ihre Erholung nachhaltig verbessern können – insbesondere dann, wenn sie auch Strategien der Grenzgestaltung erlernen (Karabinski et al. 2021). Für Unternehmen ist es daher zu empfehlen, solche Interventionen in Form von Trainings oder Coachings in das eigene Gesundheitsmanagement zu integrieren. Dies bietet für Beschäftigte die Möglichkeit, günstig, weitreichend und ohne Stigmatisierung in ihrer Grenzgestaltung und Erholung unterstützt zu werden. Für eine erfolgreiche und nachhaltige Umsetzung sollte beachtet werden, dass die Teilnahme an einer solchen Intervention für die Beschäftigten freiwillig und relevant ist, Anreize bietet und durch die Führungskräfte unterstützt wird. Es ist daher ratsam, verschiedene Formate anzubieten, z. B. sowohl mehrwöchige Trainings, die mehr als vier Trainingsstunden umfassen, als auch niedrigschwellige Kurzworkshops. Trainings und Coachings sollten sowohl in Präsenz als auch webbasiert während der Arbeitszeit angeboten werden, um möglichst viele Beschäftigte anzusprechen. Zudem ist zu empfehlen, die Umsetzung in den Alltag z. B. durch Transfercoachings zu unterstützen (Kauffeld 2016).

Darüber hinaus stellt die Unterstützung der Führungskraft einen wichtigen Einflussfaktor der Grenzgestaltung der Mitarbeitenden dar: Führungskräfte beeinflussen beispielsweise, welche Normen und Erwartungen an die Grenzgestaltung und Erreichbarkeit sich in ihrem Team entwickeln (Mazmanian 2013; Reinke und Gerlach 2021). Dies kann explizit durch entsprechende Kommunikation geschehen, aber auch implizit durch die Signalwirkung des Verhaltens der Führungskraft. Diese Erwartungen und Normen können dann nicht nur zu einer größeren Vermischung von Arbeit und Privatleben führen, sondern auch direkt die Erholung der Mitarbeitenden beeinträchtigen (Bennett et al. 2016). Es ist daher zu empfehlen, Führungskräfte in Führungskräfte-Workshops für die Wichtigkeit einer gesunden Grenzgestaltung sowie für ihre Vorbildfunktion zu sensibilisieren und gesundheitsförderliches Führungsverhalten zu trainieren. Außerdem empfiehlt es sich, Trainings zur Grenzgestaltung und Erholungsförderung für ganze Teams anzubieten. In diesen können z. B. bestehende wahrgenommene Erwartungen und Normen explizit besprochen werden. Anschließend sollte das Team gemeinsam Regelungen entwickeln, wie es die Erreichbarkeit und Grenzgestaltung zukünftig handhaben möchte, um produktiv zu bleiben, aber auch die Gesundheit der Teammitglieder aufrechtzuerhalten. Zuletzt sollten Unternehmen auch auf organisationaler Ebene Maßnahmen und Regelungen treffen, um ihre Beschäftigten vor gesundheitlichen Beeinträchtigungen zu schützen und Erholung zu fördern. So können z. B. unternehmensweite Leitlinien für die Nutzung von IKT zu Arbeitszwecken in der Freizeit vereinbart werden, um Grenzüberschreitungen in der Freizeit zu vermindern und die Erholung aller Beschäftigten zu unterstützen (Boswell et al. 2016).

Abschließend lässt sich festhalten: Eine gesundheitsgerechte Grenzgestaltung ist herausfordernd für Beschäftigte. Strategien der Grenzgestaltung und Interventionen, in denen solche Strategien erlernt werden, können dabei unterstützen, diese Herausforderung zu meistern. Für eine erfolgreiche Umsetzung liegt es jedoch auch an den Unternehmen, eine Organisationskultur zu schaffen, die eine gesunde Grenzgestaltung ermöglicht und die Notwendigkeit von Erholung – auch und gerade für die Leistungsfähigkeit – anerkennt und unterstützt.
